# Simultaneous Determination of Xanthine and Hypoxanthine Using Polyglycine/rGO-Modified Glassy Carbon Electrode

**DOI:** 10.3390/molecules28031458

**Published:** 2023-02-02

**Authors:** Ting Wang, Lin Zhang, Chengyu Zhang, Dongmei Deng, Dejia Wang, Liqiang Luo

**Affiliations:** 1 School of Health & Social Care, Shanghai Urban Construction Vocational College, Shanghai 201401, China; 2 College of Sciences, Shanghai University, Shanghai 200444, China

**Keywords:** polyglycine, reduced graphene oxide, simultaneous determination, xanthine, hypoxanthine, electrochemical sensor

## Abstract

A novel electrochemical sensor was developed for selective and sensitive determination of xanthine (XT) and hypoxanthine (HX) based on polyglycine (p-Gly) and reduced graphene oxide (rGO) modified glassy carbon electrode (GCE). A mixed dispersion of 7 μL of 5 mM glycine and 1 mg/mL GO was dropped on GCE for the fabrication of p-Gly/rGO/GCE, followed by cyclic voltammetric sweeping in 0.1 M phosphate buffer solution within −0.45~1.85 V at a scanning rate of 100 mV·s^−1^. The morphological and electrochemical features of p-Gly/rGO/GCE were investigated by scanning electron microscopy and cyclic voltammetry. Under optimal conditions, the linear relationship was acquired for the simultaneous determination of XT and HX in 1–100 μM. The preparation of the electrode was simple and efficient. Additionally, the sensor combined the excellent conductivity of rGO and the polymerization of Gly, demonstrating satisfying simultaneous sensing performance to both XT and HX.

## 1. Introduction

**Scheme 1:**
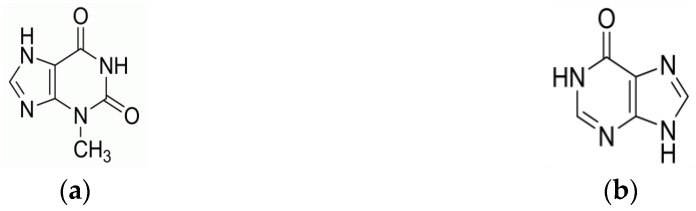
Structures of XT and HX: (**a**) XT and (**b**) HX.

In biological processes, the functional role of purine bases and their derivatives have been widely investigated for their deep connection to the biochemical reaction in the bodies [1]. As we know, purines are engaged in many metabolic procedures as cofactors and play a crucial role in fundamental bioprocesses. The changes in some pathological conditions can cause changes in purine derivatives in serum and urine, which are vital biochemical markers of some diseases [2]. As displayed in Scheme 1, xanthine (XT) and hypoxanthine (HX) are regarded as major products of deoxynucleotide metabolism and purine nucleotide, and their concentrations in serum and urine are crucial for clinical diagnoses, such as gout, hyperuricemia, leukemia, pneumonia and renal failure [3,4]. In addition, it has been associated with Parkinson’s disease when the concentration of HX in the cerebrospinal fluid of patients is lower than that of healthy individuals [5]. On the other hand, XT is an important intermediate in purine metabolism, and abnormalities can lead to xanthuria. Therefore, quantitative determinations of XT and HX are of great importance in clinical diagnosis and drug therapy.

To date, methods for the determination of XT and HX have been greatly developed, such as high-performance liquid chromatography [6,7], mass spectrometry [8] and capillary electrophoresis [9,10]. Nevertheless, these methods desire complex procedures. The electrochemical method can be an effective alternative for XT and HX sensing as a simple, cost-effective and reliable technique. The performance of an electrochemical sensor is especially based on the unique properties of the chemically modified electrode, such as excellent electrocatalytic activity and good electrical conductivity. However, from the viewpoint of clinical practice, it is still a great challenge to develop a highly sensitive, reliable and low-cost electrochemical-sensing platform for the simultaneous quantification of XT and HX.

Graphene, a desirable material for electrochemical-sensing applications, is a single-layer honeycomb lattice of carbon atoms with a high ability of electron transfer and flexibility and a large specific surface area [11,12]. Treating graphene with a strong oxidizing agent can produce graphene oxide (GO), which contains functional oxygen groups such as hydroxyl, carbonyl and carboxyl groups [13], making it hydrophilic, soluble in water and other solvents and able to form a stable solution [14,15]. However, the synthetic route of reduced graphene oxide (rGO) from GO through the chemical reduction process requires the use of overdosed toxic chemicals. Therefore, much of the literature has reported new methods for the electrochemical synthesis of rGO [16,17,18,19,20]. Compared with other graphene preparation technologies, these methods can synthesize a stable film on the surface of the electrode without any other process [21,22]. Amal Raj et al. electrochemically fabricated rGO-modified glassy carbon electrode (GCE) for the simultaneous determination of uric acid, XT, HX and caffeine in human blood serum and urine [1]. Erdogan Kablan et al. synthesized rGO on GCE through a one-step electropolymerization for simultaneous detection of uric acid, XT and HX [23].

In the last few years, polymers as a research hotspot have been widely applied in the preparation of sensors to improve the sensitivity, stability and reproducibility of quantitative analysis [24,25]. Different monomers can be used to synthesize and artificially control the conductive polymers, and different nanomaterials with unique physical and chemical properties can be doped into the formed electro-polymer composites during the polymerization process [26,27]. Mathew et al. developed a poly(amino hydroxy naphthalene sulphonic acid)-modified GCE for the simultaneous determination of XT and HX [28]. Ouyang et al. fabricated poly(amino hydroxy naphthalene sulphonic acid)-modified GCE to simultaneously determine uric acid, dopamine and ascorbic acid [29].

Polyamino acids play an especially important role in drug delivery, environment and sensors [30,31,32]. As polyamino acid, polyglycine (p-Gly) has a good electrocatalytic effect [33,34]. Gilbert et al. prepared p-Gly to determine dopamine and ascorbic acid simultaneously [35]. Thomas et al. constructed a sensor for the quantification of dopamine using a multi-walled carbon nanotube/p-Gly modified carbon paste electrode [36]. Thus, the combination of rGO and p-Gly can take advantage of both the high conductivity of rGO and the electrochemical catalytic role of p-Gly to realize the electrochemical detection of HX and XT.

In this work, by exploiting the advantages of the distinguished electrocatalytic performance of p-Gly and the good electrical conductivity of rGO, a novel high-sensitivity sensor for simultaneous electrochemical quantification of XT and HX was developed using p-Gly/rGO/GCE. The proposed sensor exhibited high sensitivity and satisfying anti-interference ability, good stability and reproducibility.

## 2. Results

### 2.1. Characteristics of p-Gly/rGO Film on GCE

**Figure 1:**
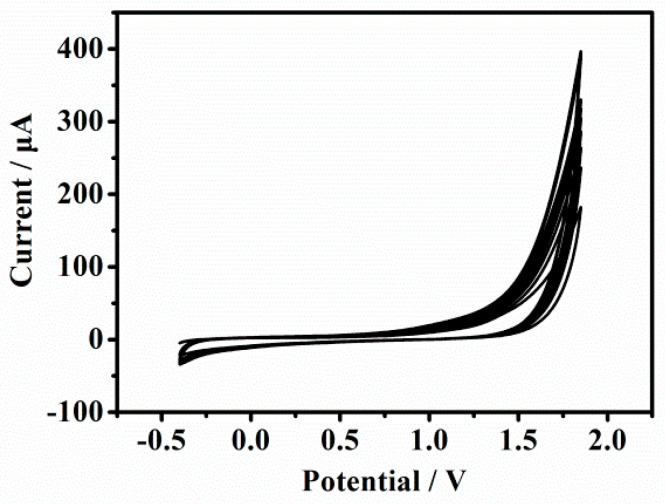
CVs of the electrochemical polymerization of p-Gly/rGO at GCE.

Cyclic voltammetry (CV) was performed in 0.1 M PBS (pH 7.0) to prepare p-Gly/rGO film on GCE. Herein, the potential range and the scanning rate are two important factors in preparing the polymer film. With the scanning rate of 100 mV·s^−1^, experimental results showed the formation of homogeneous and stable p-Gly/rGO film in −0.45~1.85 V. In Figure 1, the oxidation current gradually increased during repeated CVs, indicating that p-Gly has been successfully polymerized. The possible mechanism can be explained as follow: When the potential is applied, the surface of GCE is oxidized; At the same time, the amino group on glycine is activated into free radicals, which are connected to the electrode surface through C–N bond, and the activated amino group and the carboxyl group are condensed on the electrode surface and polymerized to form a p-Gly film [37,38].

**Figure 2:**
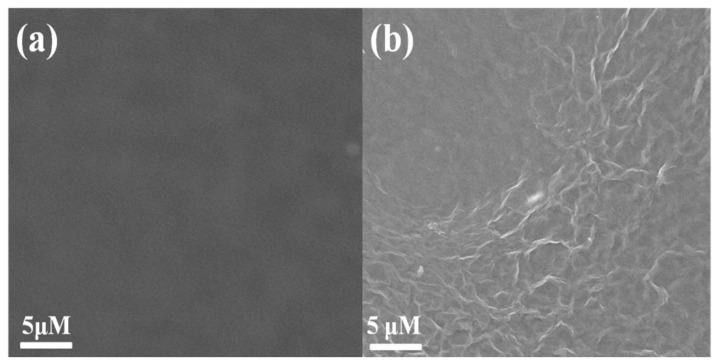
SEM images of (**a**) bare GCE and (**b**) p-Gly/rGO/GCE.

SEM is a powerful technique to characterize surface morphology. As displayed in Figure 2, compared with the SEM of bare GCE (Figure 2a), when p-Gly and rGO were deposited on the GCE surface, the p-Gly/rGO could be clearly observed, indicating that p-Gly and rGO have been successfully deposited on GCE (Figure 2b).

### 2.2. Electrochemical Behavior of XT and HX on p-Gly/rGO/GCE

**Figure 3:**
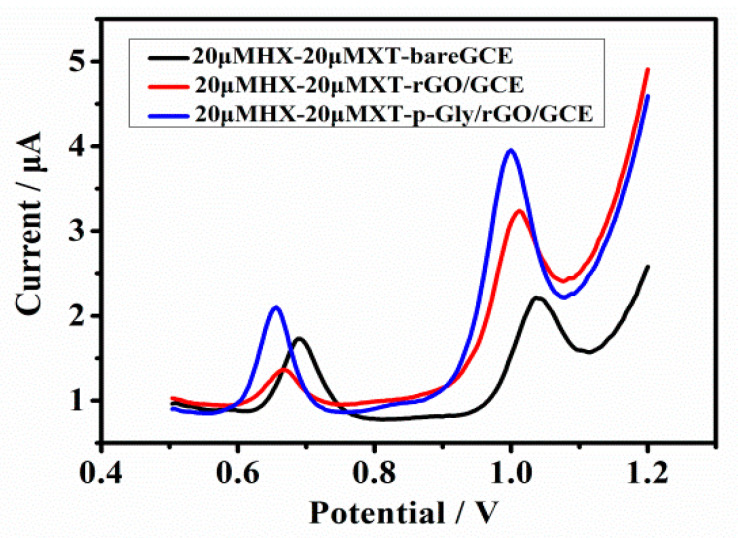
DPV curves of bare GCE, rGO/GCE and p-Gly/rGO/GCE after adding 20 μM XT and 20 μM HX in 0.1 M PBS (pH 7.0).

Figure 3 shows the differential pulse voltammetry (DPV) responses of 20 μM XT and 20 μM HX on different modified electrodes in 0.1 M PBS (pH 7.0). All DPV tests were carried out at the step potential of 0.004 V, pulse amplitude of 0.05 V and modulation time of 0.05 s. Compared with those on the bare GCE and rGO/GCE, the oxidation peak currents of XT and HX on p-Gly/rGO/GCE were significantly higher. Additionally, these two oxidation peak potentials on p-Gly/rGO/GCE were more negative than those on bare GCE, implying a decrease of overpotential for both XT and HX on p-Gly/rGO/GCE. Apparently, p-Gly displays an excellent electrocatalytic oxidation ability for the two compounds, and the electrochemical signals of XT and HX can be amplified for the large specific surface area, and excellent conductivity of rGO can accelerate the electron transfer on the electrode surface. The above experimental results confirmed that p-Gly/rGO had good electrocatalytic effects on the oxidation of XT and HX.

### 2.3. Impact of Scanning Rates

**Figure 4:**
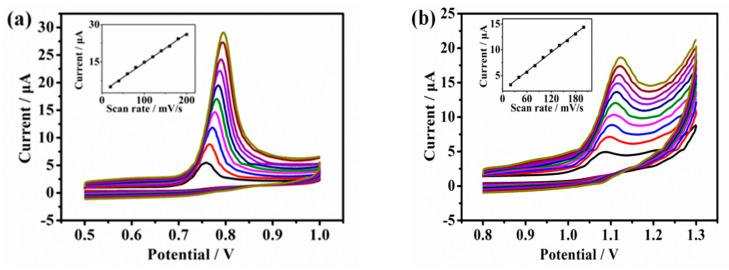
(**a**) CVs of p-Gly/rGO/GCE after adding 20 μM XT at the scanning rates of 20–200 mV·s^−1^ in 0.1 M PBS (pH 7.0). Inset: the correlated calibration plot. (**b**) CVs of p-Gly/rGO/GCE after adding 20 μM HX at the scanning rates of 20–200 mV·s^−1^ in 0.1 M PBS (pH 7.0). Inset: the correlated calibration diagram.

The peak current at different scanning rates (20–200 mV·s^−1^) was recorded by CV to study the kinetic process of XT and HX on p-Gly/rGO/GCE. Figure 4a shows the CV of 20 μM XT on p-Gly/rGO/GCE at a series of scanning rates. The oxidation peak current significantly increases with an increasing scanning rate. The figure inserted in Figure 4a is the relationship between the oxidation peak current of XT and the scanning rate. In 20–200 mV·s^−1^, the linear relationship between the oxidation peak current and the scanning rate suggested that the electrochemical reaction of XT on the electrode surface was an adsorption-control process. The linear equation is I_pa_ (μA) = 2.9250 + 0.1171 ʋ (mV·s^−1^) (R^2^ = 0.998). At the same time, Figure 4b shows the CV response curves of 20 μM HX on p-Gly/rGO/GCE at a range of scanning rates. The peak current is proportionate to the scanning rate, and the linear equation is I_pa_ (μA) = 2.105 + 0.06154 ʋ (mV·s^−1^) (R^2^ = 0.997), meaning that the electrochemical reaction of HX is also an adsorption-controlled process [14]. Additionally, with the increase of the scanning rate, the potential of the oxidation peak gradually shifted in the positive direction, denoting that the reaction process of XT and HX on p-Gly/rGO/GCE is a quasi-reversible process.

### 2.4. Optimization of the Experimental Conditions

For the best performance of p-Gly/rGO/GCE, two main experimental conditions, including the electropolymerization cycles and the pH of the electrolyte, were optimized using DPV. 

**Figure 5:**
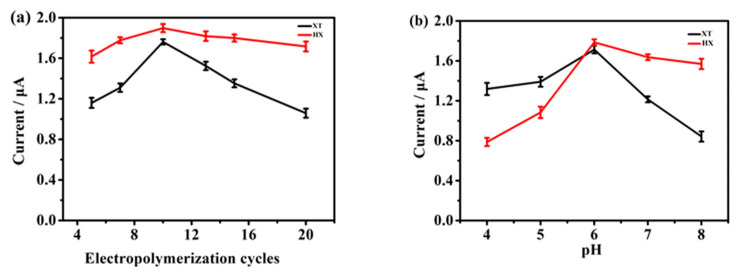
Effects of (**a**) the electropolymerization cycles and (**b**) pH values for the oxidation on p-Gly/rGO/GCE with the addition of 40 μM XT and 40 μM HX.

Polymer-modified electrodes can usually be prepared by electrochemical or chemical methods. In this work, glycine was polymerized to the electrode by electropolymerization, and the thickness of the p-Gly film was adjusted by varying the number of polymerization cycles. As demonstrated in Figure 5a, the peak current of investigated analytes reached its maximal value when the scanning cycles were 10, while the corresponding current decreased as the scanning cycles exceeded 10. Therefore, 10 cycles were selected as the best condition for the preparation of the p-Gly/rGO/GCE.

The pH of PBS can change peak current, which has a remarkable impact on the determination of XT and HX simultaneously. As displayed in Figure 5b, when the pH value varied from 4 to 8, the oxidation current increased and then decreased, reaching its maximum at pH 6.0. Thus, a pH of 6.0 was adjusted in the PBS for the subsequent experiments. Therefore, the optimal parameters of the sensor were pH 6.0 PBS and 10 cycles of polymerization.

### 2.5. Determination of XT and HX

**Figure 6:**
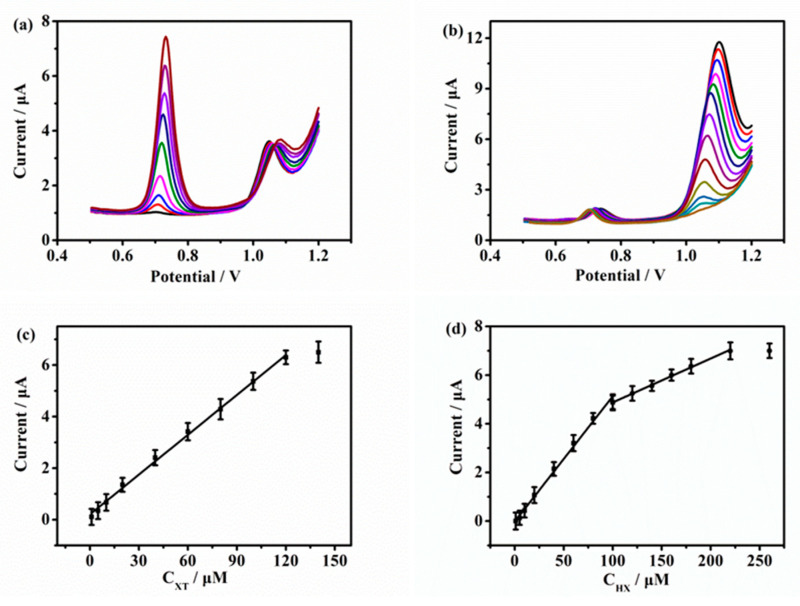
(**a**) DPVs of p-Gly/rGO/GCE with 20 μM HX for the determination of 1–120 μM XT in 0.1 M PBS (pH 6.0), (**b**) 1–220 μM HX with 20 μM XT in 0.1 M PBS (pH 6.0), (**c**,**d**) corresponding calibration plots, respectively.

DPV was used to evaluate the sensitivity of p-Gly/rGO/GCE to XT and HX under optimal conditions. Firstly, the concentration of one substance in the solution was kept constant, while the concentration of the other substance in the solution was gradually changed to measure XT and HX separately. As shown in Figure 6a, the addition concentration of XT was continuously changed (1–120 μM), and every peak was separately measured when 20 μM HX in PBS was kept constant. The results displayed that the peak current value escalated with the addition of XT and was linearly related to XT concentration. The linear equation for the relationship of current value and XT concentrations is I_pa_(μA) = 0.2046 + 0.0515C_XT_ (μM) (R^2^ = 0.997). Similar to the above process, 20 μM XT was kept constant in 0.1 M PBS (pH 6.0), and HX was separately measured in the system. As shown in Figure 6b, when HX increased from 1 to 220 μM, the peak current value continuously increased. The linear equation is I_pa_ (μA) = −0.01775 + 0.0515C_HX_ (μM) (R^2^ = 0.994) in the concentration of 1–100 μM, and I_pa_ (μA) = 3.073 + 0.018C_HX_ (μM) (R^2^ = 0.994) in the concentration of 100–220 μM.

**Figure 7:**
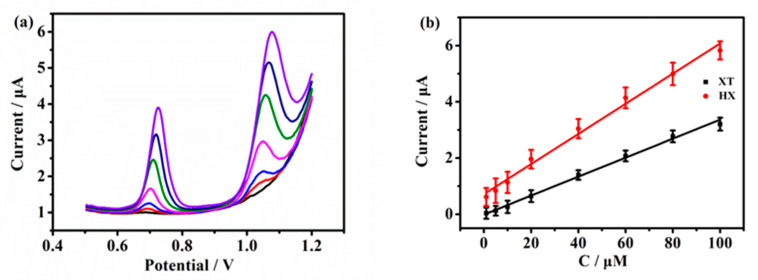
(**a**) DPVs of the p-Gly/rGO/GCE to simultaneously determine XT and HX in 0.1 M PBS (pH 6.0). (**b**) The corresponding calibration plots.

To simultaneously determine XT and HX, their concentrations in the solution under the same determination conditions were concurrently changed. With increasing concentrations of XT and HX, the peak current values on p-Gly/rGO/GCE increased. In Figure 7, the peak current of XT oxidation is linearly related to its concentration in 1–100 μM, and the equation is I_pa_ (μA) = −0.013 + 0.0338C_XT_ (μM) (R^2^ = 0.995), with a limit of detection (LOD) of 0.1 μM (S/N = 3). Furthermore, its oxidation peak current is linearly related to the content in 1–100 μM and the equation is Ipa(μA) = 0.7127 + 0.0536C_HX_ (μM) (R^2^ = 0.994), and the LOD is calculated to be 0.2 μM.

The performance comparison in detecting XT and HX between some reported modified electrodes and p-Gly/rGO/GCE in this work is listed in Table 1. Compared with previous reports, the constructed sensor shows appreciable electrochemical performance.

### 2.6. Reproducibility, Stability and Interference Tests

Under optimal conditions, the DPV technique was performed to test a mixed solution containing 20 μM XT and 20 μM HX on the same modified electrode five times, and then the differences in the currents were compared (Supplementary Table S1). After five groups of experiments, the relative standard deviation (RSD) of XT and HX was 4.31% and 5.27%, respectively, indicating the desirable reproducibility of the p-Gly/rGO/GCE.

The stability of the electrode was evaluated by measuring the current response of 20 μM XT and 20 μM HX on the same p-Gly/GO/GCE. Afterwards, the electrode was rinsed clean and stored at 4 °C for one week, and then the same electrode was used to measure the same concentrations of XT and HX again. The results show that the peak current measured was basically the same as that obtained by the first determination, implying that the p-Gly/rGO/GCE possessed acceptable stability. 

In addition, the anti-interference performance of the was investigated by adding some potentially interfering compounds during the determination of XT and HX (Supplementary Table S2). Under the same optimal conditions, 500 μM of interfering compounds that may exist in beverages, drugs and biological liquids were added into the solutions containing 20 μM XT and 20 μM HX, such as glucose, uric acid, ascorbic acid, citric acid, L-threonine and L-arginine. The recorded signals have no obvious effect on the measured object, indicating the agreeable anti-interference ability of the p-Gly/rGO/GCE.

### 2.7. Real Sample Analyses

The p-Gly/rGO/GCE was applied to simultaneously detect XT and HX in human serum to demonstrate its practicality. Usually, HX exists in the concentration range of 14–38 μM in healthy human serum [9], while the XT level is lower than 4 μM [43]. The contents of XT and HX in human serum were detected by the standard addition method. Table 2 presents the results, which indicates that the recoveries of analytes are acceptable and the p-Gly/rGO/GCE has high potential in the simultaneous detection of XT and HX in real samples.

## 3. Materials and Methods

### 3.1. Reagents and Apparatus

XT and HX were acquired from Shanghai Hanhong Technology Co., Ltd. (Shanghai, China). Ascorbic acid, GO, and uric acid were obtained from Sigma-Aldrich (USA). Gly, potassium dihydrogen phosphate (KH_2_PO_4_), sodium carbonate (Na_2_CO_3_), sodium chloride (NaCl), potassium nitrate (KNO_3_), citric acid, glucose, L-threonine, L-arginine and potassium hydrogen phosphate (K_2_HPO_4_) were purchased from Sinopharm Chemical Reagent Co., Ltd. (Shanghai, China). All reagents were analytically pure. Milli-Q water (18.2 MΩ·cm) was applied throughout the whole experiment. All electrochemical experiments were performed in 0.1 M phosphate buffer solution (PBS) at ambient temperature.

The conventional three-electrode system consists of modified electrode p-Gly/rGO/GCE as the working electrode and platinum and Ag/AgCl as the counter electrode and the reference electrode, respectively. The CV and DPV tests were conducted at the CHI660C (Shanghai CH Instruments, Shanghai, China) workstation. Scanning electron microscopy (SEM, JEOL-6700F, Tokyo, Japan) was applied to characterize the morphological features of the prepared p-Gly/rGO/GCE.

### 3.2. Preparation of p-Gly/rGO/GCE

Prior to use, Al_2_O_3_ powder (1 and 0.05 μm) was applied to polish the bare GCE (diameter, 3 mm) on the suede to obtain a mirror-smooth surface. The GCE was alternately rinsed with water and absolute ethanol. Afterwards, it was continuously swept in 0.5 M H_2_SO_4_ for 10 cycles (−1.0~1.0 V) to activate the electrode surface. Finally, the bare GCE was cleaned with ultrapure water and then kept at an ambient temperature for later use. 

5 mM Gly and 1 mg/mL GO dispersion were uniformly mixed by ultrasound. Then, 7 μL of the mixed dispersion was dropped onto the pretreated GCE. Afterwards, the electrode surface was dried and immersed into 0.1 M PBS (pH 7.0) with CV for 10 continuous sweeps in −0.45~1.85 V at 100 mV·s^−1^. Finally, the obtained p-Gly/rGO/GCE was rinsed with ultrapure water for future use.

## 4. Conclusions

In this work, p-Gly/rGO/GCE has been successfully prepared through simple electropolymerization and applied for the determination of XT and HX simultaneously. The p-Gly/rGO/GCE shows good electrocatalytic effect, reproducibility, stability and selectivity in the determination of XT and HX simultaneously due to the synergistic effect between p-Gly and rGO. Additionally, p-Gly/rGO/GCE has been utilized to simultaneously determine XT and HX in human blood serum samples, indicating its high potential in the analysis of real samples.

## Figures and Tables

**Table 1:** Comparison of determination performance of XT and HX of p-Gly/rGO/GCE with the recently published literature.

Electrode	Analyte	Linear Range (μM)	Detection Limit (μM)	Reference
rGO	XT HX	10–60 10–60	0.11 0.32	[1]
Poly(bromocresol purple)	XT HX	0.1–100 20–80	0.06 0.12	[39]
CoFe_2_O_4_/rGO	XT HX	2–10 2–10	0.650 0.506	[40]
Poly(L-arginine)/graphene	XT HX	0.1–10 0.2–20	0.05 0.1	[41]
Preanodized nontronite	XT HX	2.0–40 4.0–30	0.07 0.34	[42]
p-Gly/rGO	XT HX	1–100 1–340	0.1 0.2	This work

**Table 2:** Simultaneous determination performance of p-Gly/rGO/GCE in human blood serum.

Serum Sample	Analyte	Added (μM)	Founded (μM)	Recovery (%)
1	XT HX	20	18.7 19.1	93.5 95.5
2	XT HX	40	40.8 41.2	102 103
3	XT HX	60	58.5 60.8	97.5 101.3

## Data Availability

Data will be made available upon reasonable request.

## Supplementary Materials

The following supporting information can be downloaded at: https://www.mdpi.com/article/10.3390/molecules28031458/s1, Table S1: The reproducibility of p-Gly/rGO/GCE in the determination of mixed solution containing 20 μM XT and 20 μM HX in 0.1 M PBS (pH 6.0); Table S2: The influences on the peak currents of 20 μM XT and 20 μM HX in 0.1 M PBS (pH 6.0) at p-Gly/rGO/GCE.

## Sample Availability

Samples of the compounds are available from the authors upon reasonable request.
