# Supplementary material for: Simultaneous Determination of Xanthine and Hypoxanthine Using Polyglycine/rGO-Modified Glassy Carbon Electrode
Source: Molecules. 2023 Feb 2;28(3):1458. doi: 10.3390/molecules28031458 (PMC9922001; doi:10.3390/molecules28031458)
Supplement: Supplementary file 1 [file molecules-28-01458-s001.zip › molecules-2138449-supplementary.pdf]

Supplementary Materials

Simultaneous Determination of Xanthine and Hypoxanthine Using Polyglycine/rGO Modified Glassy Carbon Electrode

Ting Wang [1], Lin Zhang [2], Chengyu Zhang [2], Dongmei Deng [2], Dejia Wang [2], Liqiang Luo [2, *]

1 *School of Health & Social Care, Shanghai Urban Construction Vocational College, Shanghai 201401, China*

2 *College of Sciences, Shanghai University, Shanghai 200444, China*

* *Correspondence: luck@shu.edu.cn; Tel.: +86-21-66132404*

Table S1. The reproducibility of p-Gly/rGO/GCE in the determination of mixed solution containing 20 μM XT and 20 μM HX in 0.1 M PBS (pH 6.0).

**Analyte**	**Current (μA)**	**RSD (%)**
XT	0.636	4.31
	0.693	
	0.64	
	0.681	
	0.695	
HX	1.698	5.27
	1.894	
	1.725	
	1.722	
	1.876	

Table S2. The influences on the peak currents of 20 μM XT and 20 μM HX in 0.1 M PBS (pH 6.0) at p-Gly/rGO/GCE.

Interference	Concentration (μM)	Change in response (%)	
		XT	HX
K^+	500	+4.7	+5.2
Na^+		+3.2	+4.1
NO_3^-		+5.1	+3.2
Cl^-		+3.6	+4.9
SO_4^{2-}		+2.5	+3.4
CO_3^{2-}		+3.9	+3.7
L-threonine		+4.9	+4.3
L-arginine		+3.2	+4.6
Glucose		+5.7	+3.6
Citric acid		–4.2	–5.2
Ascorbic acid		+3.6	+2.2
Uric acid		+5.0	+2.5
